# Optogenetic targeting of astrocytes restores slow brain rhythm function and slows Alzheimer’s disease pathology

**DOI:** 10.1038/s41598-023-40402-3

**Published:** 2023-08-11

**Authors:** Yee Fun Lee, Alyssa N. Russ, Qiuchen Zhao, Stephen J. Perle, Megi Maci, Morgan R. Miller, Steven S. Hou, Moustafa Algamal, Zhuoyang Zhao, Hanyan Li, Noah Gelwan, Zhe Liu, Stephen N. Gomperts, Alfonso Araque, Elena Galea, Brian J. Bacskai, Ksenia V. Kastanenka

**Affiliations:** 1 Department of Neurology, MassGeneral Institute of Neurodegenerative Diseases, Massachusetts General Hospital and Harvard Medical School, Charlestown, MA 02129 USA; 2 Department of Anatomy and Neurobiology, Boston University School of Medicine, Boston, MA 02118 USA; 3 Department of Neuroscience, University of Minnesota, Minneapolis, MN 55455 USA

**Keywords:** Alzheimer's disease, Dementia

## Abstract

Patients with Alzheimer’s disease (AD) exhibit non-rapid eye movement (NREM) sleep disturbances in addition to memory deficits. Disruption of NREM slow waves occurs early in the disease progression and is recapitulated in transgenic mouse models of beta-amyloidosis. However, the mechanisms underlying slow-wave disruptions remain unknown. Because astrocytes contribute to slow-wave activity, we used multiphoton microscopy and optogenetics to investigate whether they contribute to slow-wave disruptions in APP/PS1 mice. The power but not the frequency of astrocytic calcium transients was reduced in APP/PS1 mice compared to nontransgenic controls. Optogenetic activation of astrocytes at the endogenous frequency of slow waves restored slow-wave power, reduced amyloid deposition, prevented neuronal calcium elevations, and improved memory performance. Our findings revealed malfunction of the astrocytic network driving slow-wave disruptions. Thus, targeting astrocytes to restore circuit activity underlying sleep and memory disruptions in AD could ameliorate disease progression.

## Introduction

Alzheimer’s disease (AD) causes memory impairments in the elderly^1^. This neurodegenerative disorder is marked by extracellular accumulation of amyloid-beta (Aβ) and intracellular aggregation of hyperphosphorylated tau in neurons^2–4^. Deposition of amyloid plaques and neurofibrillary tangles leads to neuronal cell death^5–8^, synaptic density loss^9,10^, and circuit dysfunction leading to cognitive as well as memory impairments in patients with AD^11–14^.

Additionally, sleep disturbances manifest early in the disease progression. Disruptions in deep non-rapid eye movement (NREM) slow-wave sleep (SWS) are evident in patients with mild AD^15,16^ and in individuals with mild cognitive impairment (MCI)^17^. The cortico-thalamic circuit generates slow waves as oscillations alternating between DOWN states, periods of relative quiescence, and UP states, periods of vigorous neuronal activity^18–20^. These slow oscillations, or slow waves of < 1 Hz, are prevalent during NREM sleep^21^ and are disrupted in patients with AD^22^ as well as in individuals with MCI^17^. Aβ is associated with decreased slow-wave activity (SWA) in asymptomatic adults, and reduced NREM SWA is correlated with increased Aβ deposition in the medial prefrontal cortex^23^. Reduced slow-wave power is also associated with tau pathology^15^. Thus, disturbances in SWA present a potential early biomarker for AD^24,25^.

Slow-wave disruptions observed in AD are recapitulated in mouse models of beta-amyloidosis, in which slow-oscillation power is downregulated by Aβ in young APP/PS1 (APP) mice^26^. Moreover, disruptions in slow oscillations actively contribute to the disease progression^24,26,27^. Slow oscillations play a critical role in consolidation of declarative memories during deep NREM sleep^28–33^. Slow oscillations drive the reactivation of short-term hippocampal memories by synchronizing spindle activity in the thalamus, thereby contributing to the long-term plastic changes in neocortical networks and supporting the consolidation of long-term memory in neocortex^34,35^. Boosting slow waves enhances memory performance^30^, while disruptions of slow oscillations are linked to memory impairments^23^. Similarly, memory function is impaired in APP mice^36–38^. Since slow oscillations play an important role in declarative-memory consolidation during sleep, therapeutic targeting of slow oscillations could restore sleep and memory deficits as well as slow AD progression.

Little is known about the underlying mechanisms that cause the disruption of slow waves. One of the possible mechanisms is, as Aβ targets synapses, alterations of excitatory and inhibitory neurotransmission contribute to neuronal circuit dysfunctions^26,39,40^. Both excitatory and inhibitory neurons participate in generation of slow oscillations^18,20,41–45^. Besides neurons, astrocytes actively contribute to circuit function^46,47^. Astrocytes form the tripartite synapses with pre- and post-synaptic neuronal compartments to regulate synaptic transmission via astrocytic calcium signaling^48–50^. Yet, whether astrocytes contribute to the disruption of slow oscillations in APP mice remains unclear.

Astrocytes are electrically silent cells that exhibit calcium transient elevations in response to neuronal stimulation^51^. Importantly, astrocytes actively participate in SWA and they are implicated in SWA^52^. In addition, astrocytic calcium transients are synchronized to the neuronal UP states of slow oscillations in healthy anesthetized rats^53^. In addition, more astrocytes than neurons are active during the initiation of SWA, indicating that astrocytes participate in the build-up of slow waves^53^. Electrical stimulation of individual astrocytes increases the activity of the local astrocytic networks and triggers the synchronous UP state transitions in neighboring neurons within somatosensory cortex^54^.

Elevations in resting astrocytic calcium levels were reported in APP mice^55,56^, suggesting that disruption of astrocytic activity may contribute to the cortico-thalamic circuit dysfunction driving slow waves in AD. Therefore, understanding the role of astrocytes in the disruption of cortical SWA is important. Here, we hypothesized that aberrant astrocytic activity contributes to the disruption of the cortico-thalamic circuit function, such that restoration of astrocytic function will slow AD progression in APP mice. To test this hypothesis, we monitored the calcium transients in individual astrocytes using high-resolution multiphoton microscopy in vivo. We discovered the power of astrocytic calcium transients was decreased in young APP mice. Importantly, optogenetic targeting of astrocytes restored slow oscillations, decreased amyloid deposition, normalized neuronal calcium homeostasis and rescued sleep-dependent memory function in APP mice. Our findings provided evidence for aberrant astrocytic activity contributing to slow wave disruptions. Thus, astrocytic targeting of SWA emerges as a novel therapeutic target for the prevention or treatment of AD.

## Results

### Astrocytic calcium transients associated with slow oscillations are disrupted in young APP/PS1 (APP) mice

**Figure 1:**
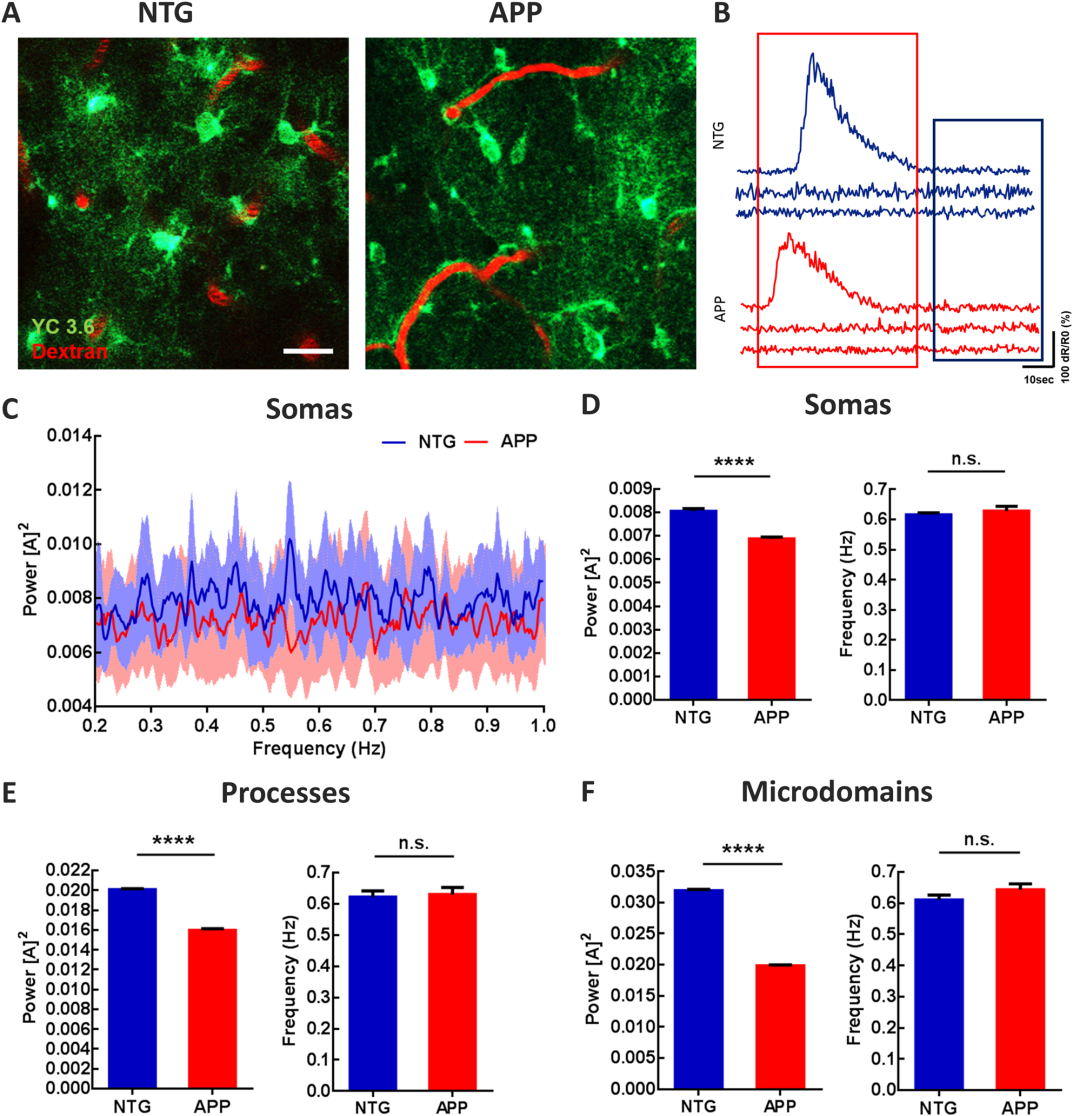
Astrocytic calcium transients are impaired at the slow-wave frequency in young APP mice. (**A**) Multiphoton microscopy image of YC3.6-expressing astrocytes in 4–6 months old NTG and APP mice. YC3.6 was expressed in the somas, processes, and endfeet of astrocytes (green), while dextran red filled the vasculature (red). (**B**) Representative ΔR/R_0_ traces of individual astrocytic calcium transients in NTG and APP mice. (**C**) Power spectral densities of calcium transients within astrocytic somas. Bright blue and red traces are averages, faint blue and red bands are SEM. (**D**) The mean power and frequency of somal calcium transients in NTG and APP mice. (**E**) The mean power and frequency of calcium transients within astrocytic processes in NTG and APP mice. (**F**) The mean power and frequency of calcium transients within astrocytic microdomains in NTG and in APP mice. Values are mean ± SEM, scale bar, 10 μm. ****p < 0.0001.

To determine whether astrocytic calcium signaling was altered in 4–6 months-old APP/PS1 (APP) mice, we monitored calcium transients within individual cortical astrocytes using imaging with multiphoton microscopy. We targeted the genetically encoded calcium indicator Yellow Cameleon 3.6 (YC3.6) to astrocytes in the somatosensory cortex via the GFAP promoter, since cortical slow oscillations traverse somatosensory cortex^57^ and slow oscillations are disrupted when imaged in somatosensory cortex^26^. YC3.6 expression was visualized in the somas, processes, and microdomains of astrocytes in non-transgenic control (NTG) and APP mice (Fig. 1A). YC3.6 is a ratiometric (R) probe. Representative ΔR/R_0_ traces are shown in Fig. 1B. Here we focused the analyses on the low amplitude, high frequency astrocytic calcium transients that were implicated in slow oscillations^53^ (Fig. 1B, blue box), not large amplitude, low frequency astrocytic calcium transients^55^ (Fig. 1B, red box). Next, we used Fourier transform analysis to characterize the astrocytic calcium transients. The percentage of astrocytes cycling at the slow-wave frequency (0.2–1 Hz) was high and did not differ significantly between APP and NTG mice (Supplemental Fig. 1, 72.11 ± 3.01% of astrocytes in NTG vs. 70.15 ± 2.93% of astrocytes in APP mice; n = 300 cells in 7 NTG mice, n = 282 cells in 7 APP mice). The average power spectral density plots of somatic calcium transients for NTG (n = 300 cells in 7 mice) and APP mice (n = 282 cells in 7 mice) are shown in Fig. 1C. The calcium transient power, which is proportional to the square of the amplitude [A]^2^, at the slow-wave frequency was significantly lower in astrocytic somas, processes and microdomains of APP mice compared to that in NTG controls (Fig. 1D, 0.0080 ± 0.00012 for NTG vs. 0.0069 ± 0.00007 for APP; Fig. 1E, 0.020 ± 0.00008 for NTG vs. 0.016 ± 0.00015 for APP; Fig. 1F, 0.032 ± 0.00022 for NTG vs. 0.020 ± 0.00015 for APP; ****p < 0.0001). The frequency at peak power remained comparable in astrocytic somas, processes, and microdomains across genotypes (Fig. 1D, 0.62 ± 0.006 for NTG versus 0.63 ± 0.016 for APP; Fig. 1E, 0.62 ± 0.02 for NTG vs. 0.63 ± 0.022 for APP; Fig. 1F, 0.61 ± 0.016 for NTG vs. 0.064 ± 0.019 for APP). Thus, astrocytic calcium transients associated with SWA are disrupted in APP mice.

### Optogenetic activation of cortical astrocytes restores slow waves in young APP mice

**Figure 2:**
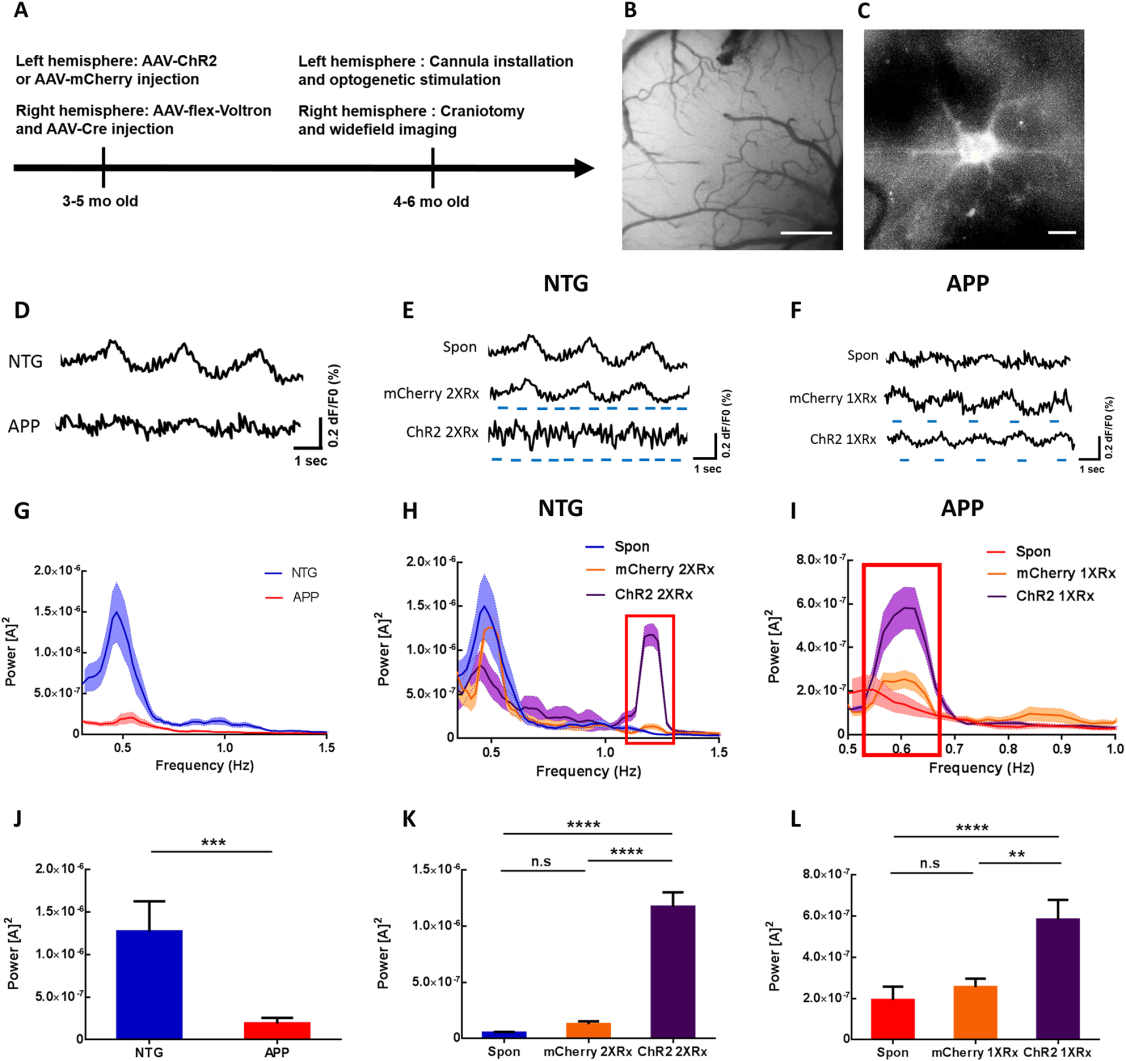
Optogenetic targeting of astrocytes allows manipulation of slow oscillations. (**A**) Experimental design. (**B**) Low resolution wide-field image of Voltron fluorescence. Scale bar, 500 μm. (**C**) High resolution wide-field image of a neuron expressing Voltron. Scale bar, 10 μm. (**D**) Representative traces of voltage sensor signal in NTG and APP mice. (**E**) Representative traces of voltage sensor signal acquired spontaneously (Spon), during light activation of mCherry lacking ChR2 at 1.2 Hz (mCherry 2XRx), or during optogenetic activation of ChR2 at 1.2 Hz (ChR2 2XRx) in NTG mice. Light pulse stimulations are shown in blue. (**F**) Representative traces of voltage sensor signal acquired spontaneously (Spon), during light activation of mCherry lacking ChR2 at 0.6 Hz (mCherry 1XRx), or during optogenetic activation of ChR2 at 0.6 Hz (ChR2 1XRx) in APP mice. Light pulse stimulations are shown in blue. (**G**–**I**) Power spectral density plots of slow oscillations in NTG (H) and APP (**I**) mice across conditions. Mean ± SEM. (**J**) Bar graph comparing the average power of slow oscillations in NTG and APP mice. (**K**) Bar graph comparing the average power of slow oscillations in NTG mice across conditions. (**L**) Bar graph comparing the average power of slow oscillations in APP mice across conditions. **p < 0.01, ***p < 0.001, ****p < 0.0001.

We had previously shown that APP mice exhibit disruptions in SWA compared to NTG controls measured with voltage sensitive dyes^26^. The power of slow waves was significantly lower in APP mice compared to NTG mice starting at 3 months of age, while the frequency remained comparable at 0.6 Hz^26^. Since astrocytes actively contribute to slow oscillations and astrocytic calcium transients associated with slow waves are disrupted, we tested whether optogenetic activation of astrocytes would restore neuronal slow oscillations in APP mice. 3–5 months-old APP and NTG mice received viral delivery of ChannelRhodopsin2-mCherry (ChR2) or mCherry lacking ChR2. Viral vectors targeted astrocytes via the GFAP promoter in the left hemisphere (Fig. 2A).

In addition to ChR2 or mCherry injections in the left hemisphere, animals received viral injections of a voltage sensor Voltron and AAV9-hsyn-Cre into the contralateral somatosensory cortex (Fig. 2A). Subsequently, a cannula was implanted over the ChR2/mCherry injection site and a 5 mm cranial window was installed over the right somatosensory cortex expressing Voltron (Fig. 2B,C). Cortical slow waves were monitored using wide-field microscopy, before and during light activation of ChR2/mCherry-expressing astrocytes (Fig. 2D–F). Since slow oscillations propagate as traveling waves, optogenetic stimulation in anterior left cortex was expected to activate the local circuit that would then propagate the wave into the contralateral hemisphere, where slow waves would be monitored with voltage sensors.

Neuronal population imaging using Voltron revealed disruptions of slow oscillations in APP mice, consistent with earlier reports (Fig. 2D)^26^. To determine whether optogenetic targeting of astrocytes was able to drive slow oscillations, we stimulated astrocytes at twice the endogenous frequency of slow waves, 1.2 Hz, in NTG mice. This allowed us to distinguish between endogenous slow oscillations and optogenetically elicited slow waves. Optogenetic targeting of astrocytes elicited slow waves at twice the endogenous frequency (Fig. 2E Spon v.s. ChR2 2XRx). However, light stimulation of mCherry in absence of ChR2 failed to do so (Fig. 2E Spon v.s. mCherry 2XRx). Furthermore, light activation of ChR2-expressing astrocytes at the endogenous frequency of slow waves, 0.6 Hz, restored the SWA in APP mice (Fig. 2F Spon v.s. ChR2 1XRx). Yet, light activation of mCherry in absence of ChR2 failed to do so (Fig. 2F Spon v.s. mCherry 1XRx).

Fourier transform analysis was performed to elucidate the power–frequency relationships. The power spectral densities of slow oscillations were plotted using Matlab (Fig. 2G–I). Endogenous slow waves oscillated spontaneously at 0.4–0.6 Hz in NTG and APP mice (Fig. 2G)^26^. Fourier transform analysis revealed decreases in the power of spontaneous slow waves in APP mice compared to NTG group (Fig. 2G,J; n = 37 traces in 6 NTG mice, n = 30 traces in 6 APP mice). The slow-wave power was significantly lower in young APP mice (Fig. 2J; 1.27 × 10^–6^ ± 3.54 × 10^–7^ for NTG vs. 1.93 × 10^–7^ ± 6.48 × 10^–8^ for APP; ***p < 0.001). A spectral power density plot revealed a pronounced peak around 1.2 Hz in NTG mice whose astrocytes were optogenetically driven at 1.2 Hz (ChR2 2XRx), but not in mCherry-expressing (mCherry 2XRx) controls (Fig. 2H, red box). The power at 1.2 Hz increased significantly during optogenetic stimulation, but not light stimulation of mCherry (Fig. 2K; 5.19 × 10^–8^ ± 7.45 × 10^–9^ for Spon vs. 1.29 × 10^–7^ ± 2.56 × 10^–8^ for mCherry 2XRx vs .1.17 × 10^–6^ ± 1.28 × 10^–7^ for ChR2 2XRx; ****p < 0.0001) (Fig. 2H,K; NTG Spontaneous: n = 37 traces in 6 mice, NTG mCherry 2XRx: n = 61 traces in 5 mice, NTG ChR2 2XRx: n = 40 traces in 5 mice). This suggested that optogenetic stimulation of cortical astrocytes was able to drive SWA at twice the endogenous frequency in NTG mice.

We then determined whether optogenetic targeting of astrocytes had an effect on slow-oscillation power in anesthetized APP mice. Optogenetic stimulation of ChR2-expressing astrocytes at the endogenous frequency of slow waves, 0.6 Hz, restored the power of slow waves in APP mice (Fig. 2I, red box, L; 1.93 × 10^–7^ ± 6.48 × 10^–8^ for Spon vs. 2.56 × 10^–7^ ± 4.07 × 10^–8^ for mCherry 1XRx vs. 5.82 × 10^–7^ ± 9.63 × 10^–8^ for ChR2 1XRx; **p < 0.01; ****p < 0.0001) (Fig. 2I, L; APP Spontaneous: n = 30 traces in 6 mice, APP mCherry 1XRx: n = 45 traces in 5 mice, APP ChR2 1XRx: n = 53 traces in 5 mice). Thus, the SWA was restored following light activation of astrocytes at the endogenous frequency of slow waves.

To determine whether optogenetic stimulation of astrocytes during slow wave sleep (SWS) impacted the cortical local field potential (LFP), mice underwent injection of ChR2 in the left hemisphere and implantation of a stereotrode array in the contralateral hemisphere, targeting the region imaged with Voltron. The cortical LFP was recorded as animals slept and underwent optogenetic stimulation, and SWS was identified using the theta delta ratio in the setting of prolonged immobility in a sleep posture. Compared to baseline LFP, optogenetic stimulation of ChR2-expressing astrocytes at 0.6 Hz was associated with a small but significant increase in LFP power at 0.6 Hz (n = 3 mice, *p* = 0.030, 1-tailed paired t-test; Supplemental Fig. 1A–C). These results indicated that optogenetic targeting of astrocytes could be used to successfully manipulate neuronal slow oscillations in APP mice.

To verify whether the ChR2 and mCherry targeted astrocytes and not other brain cells, we performed immunohistochemical analyses of the localization of mCherry with astrocytic (GFAP), neuronal (NeuN) and microglial (Iba1) markers in post-mortem brain tissue (Supplemental Fig. 3). mCherry-expressing cells colocalized with GFAP + positive cells (Supplemental Fig. 3E; 92.74 ± 1.21% of cells were GFAP+ ; 825/891 cells from 10 sections in 5 mice), not NeuN (Supplemental Fig. 3F; 98.43 ± 0.47% cells were NeuN-; 877/891 cells from 10 sections in 5 mice), nor Iba1 (Supplemental Fig. 3K; 98.24 ± 0.52% of mCherry-expressing cells lacked Iba-1; 421/431 cells from 10 sections in 6 mice). Thus, the GFAP promoter targeted ChR2-mCherry or mCherry specifically to astrocytes, not neurons or microglia.

### The rhythmic optogenetic stimulation of astrocytes decreases amyloid beta deposition

**Figure 3:**
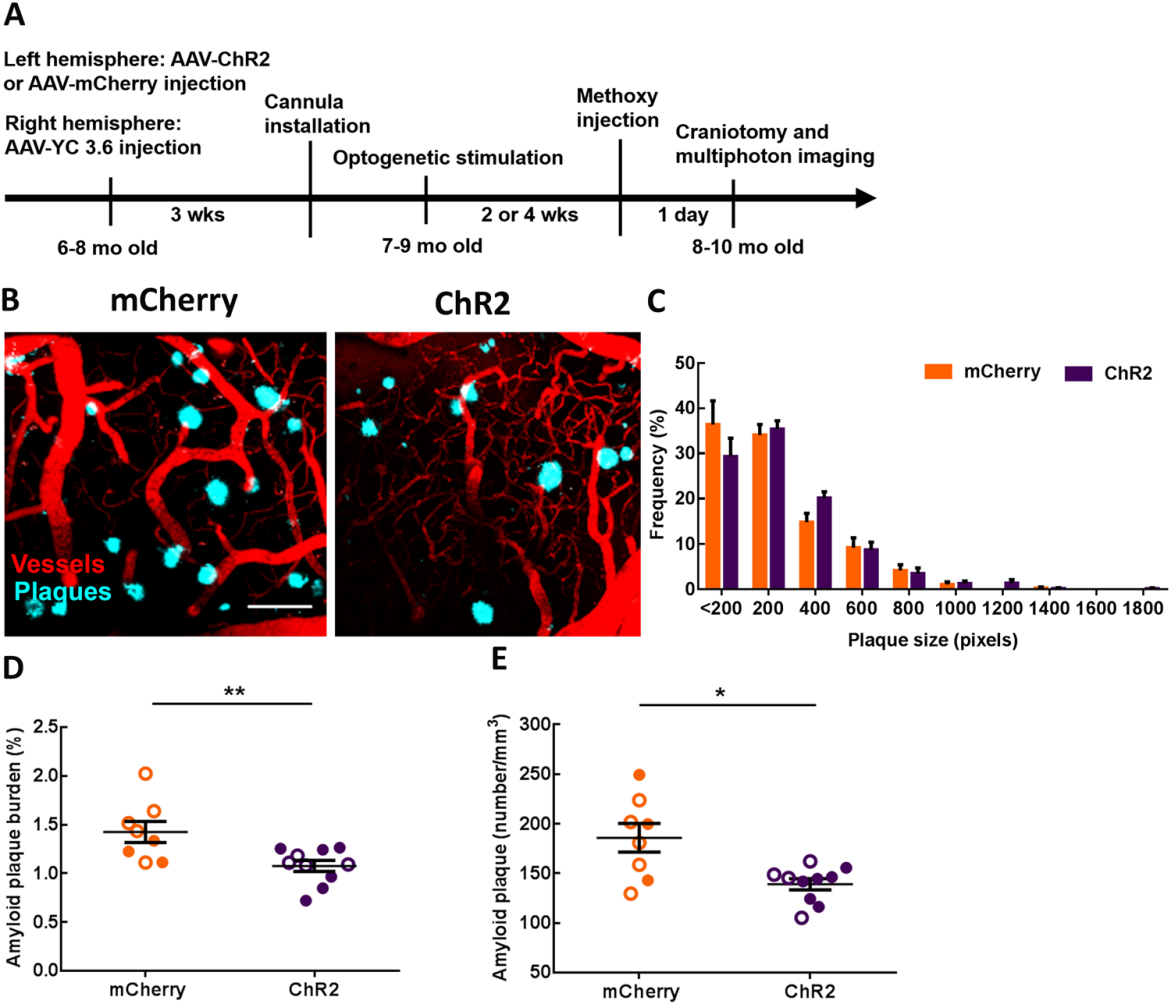
Slow-wave rescue decreases amyloid plaque deposition in APP mice. (**A**) Experimental design. (**B**) Representative multiphoton images of methoxy-X04 positive amyloid plaques after light activation of cortical astrocytes in mCherry and ChR2 groups. Plaques are shown in blue. Dextran red filled blood vessels are in red. (**C**) The frequency distribution of amyloid plaque size in mCherry and ChR2 groups. (**D**) Amyloid plaque burden across conditions. (**E**) Amyloid plaque number across conditions. Values are presented as mean ± SEM. Males are represented by closed circles, and females by open circles. Scale bar, 100 μm, *p < 0.05, **p < 0.01.

Since optogenetic targeting of astrocytes restored slow waves, and SWA actively contributed to the clearance of Aβ^58^, we next determined whether optogenetic restoration of SWA via astrocytes could slow the accumulation of Aβ in APP mice. To that end, ChR2- or mCherry-expressing cortical astrocytes were stimulated with light at 0.6 Hz, 24 h per day continuously for 2 or 4 weeks in aged APP mice. We decided to stimulate during the day and night, since prior work showed APP mice exhibiting deficits in NREM sleep during the day and night^59,60^. At the end of the light treatment, cranial windows were installed over the right posterior cortex, contralateral to the ChR2/mCherry site. High-resolution multiphoton microscopy allowed monitoring of methoxy-X04-positive amyloid plaques after treatment in vivo (Fig. 3A). Multiphoton images revealed the presence of amyloid plaques in 8–10 months-old APP mice (Fig. 3B). Restoring slow oscillations decreased amyloid deposition in the ChR2 group compared to mCherry controls (Fig. 3B). The frequency distribution of plaque size is shown in Fig. 3C. The size of the plaques was not significantly different across conditions (Fig. 3C). However, amyloid plaque burden, which takes into account plaque numbers and size, was significantly lower after optogenetic treatment compared to light activation of mCherry (Fig. 3D; 1.43 ± 0.11% burden for mCherry vs. 1.01 ± 0.06% burden for ChR2; mCherry: n = 57 fields of view (FOV) in 8 APP mice; ChR2: n = 82 FOV in 10 APP mice; **P < 0.01). Also, the number of amyloid plaques was significantly lower in the ChR2 group compared to that in mCherry controls (Fig. 3E; 185.9 ± 14.44 plaques/mm^3^ for mCherry vs. 139.0 ± 5.69 plaques/mm^3^ for ChR2; mCherry: n = 57 FOV in 8 APP mice; ChR2: n = 82 FOV in 10 APP mice; *P < 0.05). Therefore, astrocyte-dependent rescue of slow waves reduced the rate of amyloid plaque deposition in APP mice. Random optogenetic stimulation of astrocytes failed to significantly alter amyloid plaque number or amyloid plaque burden in APP mice compared to non-treated APP mice (Supplemental Fig. 4A,B).

**Figure 4:**
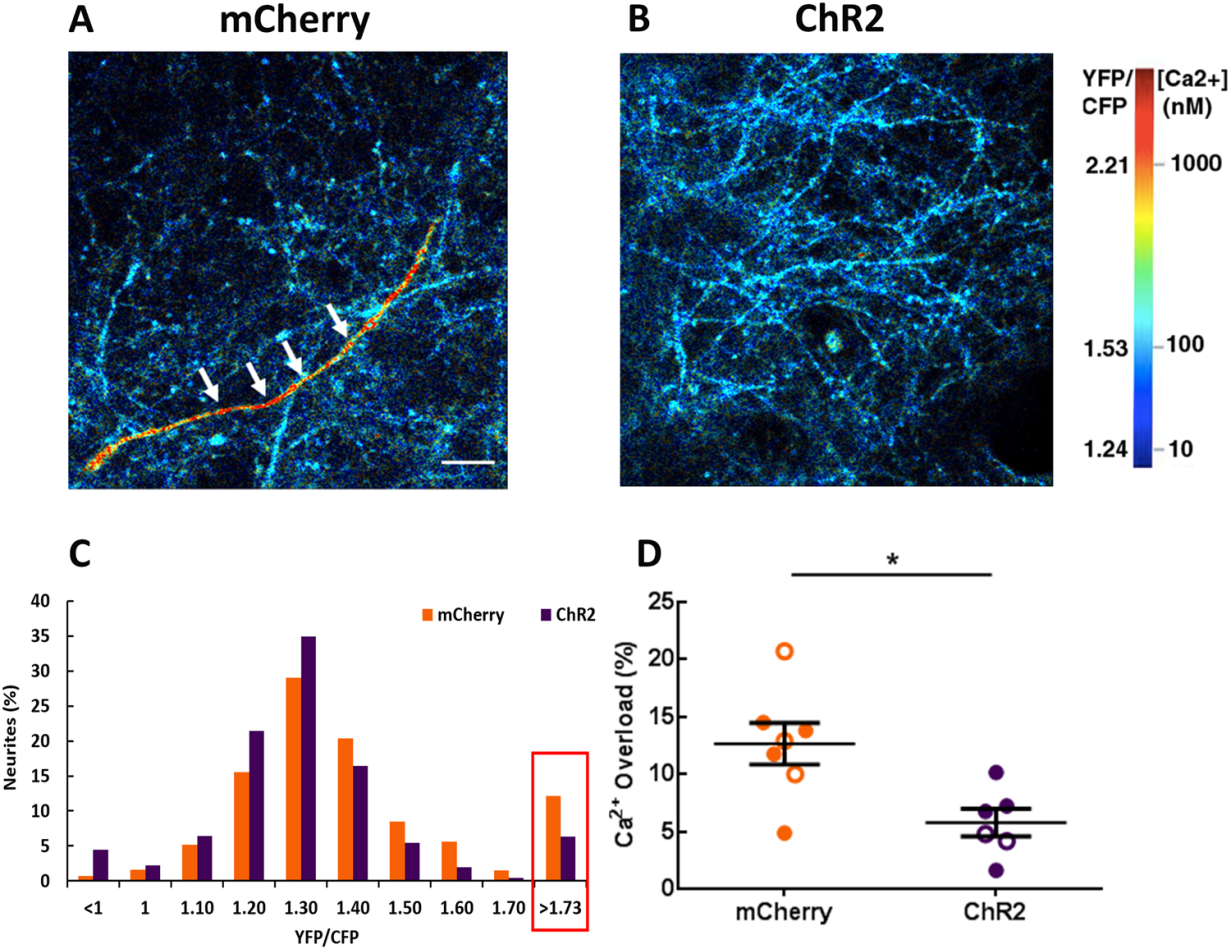
Optogenetic rescue normalizes neuronal calcium in APP mice. (**A**, **B**) The images pseudocolored according to the intraneuronal calcium concentration, acquired from APP mice expressing mCherry (**A**) or ChR2 (**B**). A neuronal process exhibiting calcium overload is seen in red (white arrows). (**C**) Histogram showing the distribution of YFP/CFP ratios in neurites expressing YC 3.6. Neuronal calcium overload was defined as a YFP/CFP ratio larger than 2 standard deviations above the average YFP/CFP ratio in the neurons of NTG mice. The ratio of YFP/CFP > 1.73 was considered as calcium overload. (**D**) The percentage of neurites exhibiting calcium overload after 2–4 week light activation of astrocytes. Values are mean ± SEM. Males are represented by closed circles and females by open circles. Scale bar, 100 μm, *p < 0.05.

### The rhythmic optogenetic stimulation of astrocytes normalizes neuronal calcium levels in APP mice

Intracellular calcium levels are tightly regulated for healthy neuronal function. APP mice exhibit disruptions in neuronal calcium homeostasis that result in elevated resting calcium levels, or calcium overload^61,62^. Calcium levels were monitored using multiphoton microscopy after 2–4 weeks of optogenetic stimulation. The genetically encoded ratiometric calcium reporter YC3.6 under the CBA promoter was used to target cortical neuronal processes, neurites. YC3.6 allows the determination of absolute calcium concentration in neurons, as well as dynamic changes. We determined whether light activation of ChR2-expressing astrocytes would alter neuronal calcium levels in APP mice. Thus, neuronal calcium was used as a functional readout of treatment efficacy. While the majority of neuronal processes, or neurites, exhibited normal calcium levels in APP mice (Fig. 4A, blue neurons), some neurites exhibited abnormally high calcium (Fig. 4A, red neuron, white arrows). Restoration of slow waves led to a decrease in the percentage of neurons with calcium overload (Fig. 4B). A YC3.6 ratio greater than two standard deviations above the mean value (> 1.73) in NTG neurons was defined as calcium overload (Fig. 4C red box)^26,27,61,62^. The percentage of neuronal processes exhibiting calcium overload was lower in the ChR2 group compared to the mCherry group (Fig. 4 C, D; 12.65 ± 1.8% for mCherry vs. 5.79 ± 1.2% for ChR2; mCherry: n = 2182 neurites in 23 FOV of 7 mice; ChR2: n = 2014 neurites in 24 FOV of 6 mice; *p < 0.05). Thus, astrocyte-dependent rescue of slow waves decreased neuronal calcium overload and restored neuronal calcium homeostasis in APP mice.

### The rhythmic optogenetic stimulation in astrocytes restores sleep-dependent memory performance in APP mice

**Figure 5:**
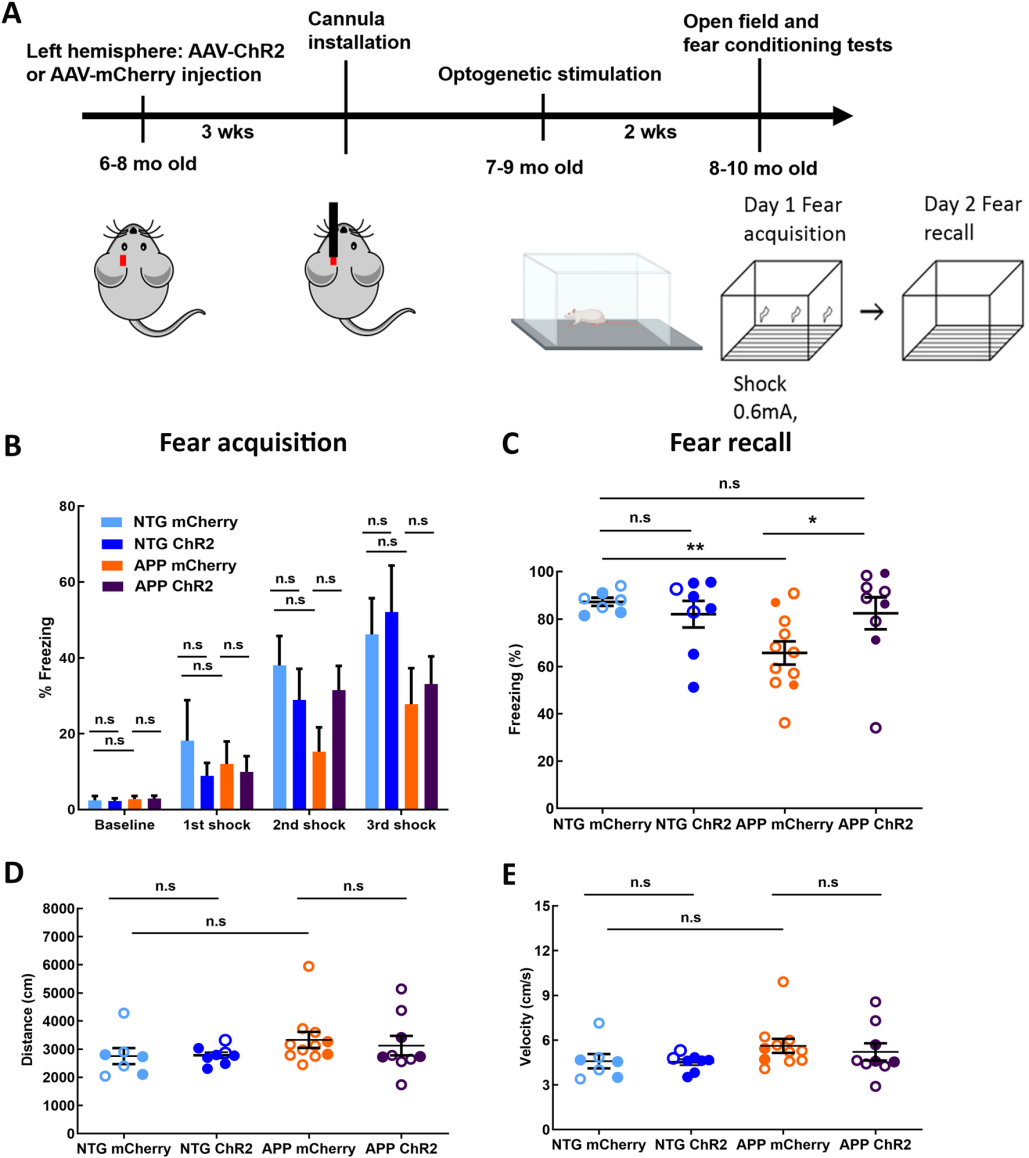
Optogenetic activation of astrocytes at the slow-wave frequency improves sleep-dependent memory performance in APP mice. (**A**) Experimental design. (**B**) Percentage of time spent freezing before and during fear conditioning. (**C**) Percentage of time spent freezing during fear recall. Sleep-dependent memory performance was assessed across groups. (**D**) The total distance traveled during open field test across conditions. (**E**) The average velocity of movement during open field test across conditions. Values are mean ± SEM. Males are represented by closed circles, and females by open circles. n.s non-significant, *p < 0.05, **p < 0.01.

To determine whether optogenetic targeting of astrocytes enhanced memory consolidation, we subjected NTG controls and APP mice to contextual fear conditioning test (Fig. 5A). Fear acquisition was performed on day 13 of light treatment. The freezing levels at baseline were similar in all four groups (Fig. 5B; 2.44 ± 1.13% for NTG mCherry vs. 2.27 ± 0.76% for NTG ChR2, 2.77 ± 0.79% for APP mCherry vs. 2.90 ± 0.77% for APP ChR2). Delivery of 3 consecutive electric shocks resulted in progressive increases in freezing. Additionally, the freezing levels after the delivery of first, second and third shocks were comparable between respective mCherry and ChR2 groups (Fig. 5B; 1st shock: 18.17 ± 10.7% for NTG mCherry vs. 8.86 ± 3.67% for NTG ChR2, 12.04 ± 5.93% for APP mCherry vs. 9.97 ± 4.13% for APP ChR2; 2nd shock: 38.04 ± 7.81% for NTG mCherry vs. 28.97 ± 8.75% for NTG ChR2, 15.25 ± 6.48% for APP mCherry vs. 31.52 ± 6.41% for APP ChR2; 3rd shock: 46.23 ± 9.54% for NTG mCherry vs. 52.16 ± 13.03% for NTG ChR2, 27.82 ± 9.49% for APP mCherry vs. 33.12 ± 7.32% for APP ChR2). Thus, fear acquisition was not significantly altered by optogenetic treatment of APP mice. Following fear acquisition, the mice were allowed to sleep and consolidate their memories overnight during light activation of ChR2 or mCherry. Fear recall was tested the following day. The contextual fear memory was impaired in APP mice (Fig. 5C; 87.29 ± 1.67% for NTG mCherry vs. 65.73 ± 4.92% for APP mCherry; **p < 0.01). The freezing levels were significantly higher in APP ChR2 group compared to that in APP mCherry group (Fig. 5C; 65.73 ± 4.92% for APP mCherry vs. 82.46 ± 6.73% for APP ChR2; *p < 0.05). In addition, the freezing levels were comparable between NTG mCherry group and APP ChR2 group (Fig. 5C; 87.29 ± 1.67% for NTG mCherry vs. 82.46 ± 6.73% for APP ChR2), indicating that optogenetic treatment restored sleep-dependent memory consolidation in APP mice to that in NTG littermates. Optogenetic treatment of NTG mice failed to further improve memory (Fig. 5C; 87.29 ± 1.67% for NTG mCherry vs. 82.09 ± 5.99% for NTG ChR2).

The same mice were subjected to a locomotor test to determine whether increases in freezing could be attributed to deficits in locomotor activity. The distance travelled in the open field was not significantly different for the four groups (Fig. 5D; 2754 ± 285.3 cm for NTG mCherry vs. 2785 ± 110.2 cm for NTG ChR2, 3329 ± 285 cm for APP mCherry vs. 3125 ± 346.1 cm for APP ChR2). The average velocity of the APP mice did not show significant group differences (Fig. 5E; 4.59 ± 0.48 cm/s for NTG mCherry vs. 4.52 ± 0.20 cm/sfor NTG ChR2, 5.61 ± 0.47 cm/s for APP mCherry vs. 5.21 ± 0.58 cm/s for APP ChR2). Our data indicated that the locomotion of the APP mice was not impaired by optogenetic treatment. Thus, slow wave rescue via optogenetic targeting of astrocytes improved sleep-dependent memory consolidation in APP mice.

We subsequently verified that optogenetic stimulation of astrocytes did not result in neurodegeneration in APP mice (Supplemental Fig. 5). Thus, blue light did not result in significant phototoxicity. In addition, the protein levels of aquaporin4 (AQP4) in GFAP+ astrocytes were not significantly different between groups (Supplemental Fig. 6), which indicated that optogenetic stimulation of astrocytes did not significantly alter the AQP4 expression in the astrocytes. The phospho-tau levels were examined in APP mice expressing ChR2 or mCherry after light treatment. The percentage of phospho-tau-positive area in the cortex was calculated. Optogenetic activation of astrocytes did not significantly alter the levels of phosphorylated tau in APP/PS1 mice (Supplemental Fig. 7). Similarly, optogenetic stimulation of astrocytes did not significantly alter the c-Fos expression in APP/PS1 mice (Supplemental Fig. 8).

## Discussion

Patients with AD exhibit memory impairments and sleep disruptions. Slow oscillations, important for memory consolidation during sleep, are impaired in patients with AD and mouse models of beta-amyloidosis. Here we determined whether astrocytes contribute to slow-wave disruption in APP mice.

It is increasingly recognized that astrocytes, in addition to modulating local synaptic plasticity, control the activity of neural networks. Astrocytes actively participate in circuit function by interacting with pre- and post-synaptic neurons via tripartite synapses^48–50^ and by communicating with neighboring astrocytes via gap junctions^63–65^. As each individual astrocyte interacts with a multitude of neurons, astrocytes exhibit calcium transients indicative of the activity of the circuit in which that astrocyte is participating. We monitored astrocytic calcium transients of low amplitude and high frequency (0.02–1 Hz). We discovered decreases in the power of calcium transients in astrocytes concomitant to aberrant slow oscillations in young APP mice. This finding was in contrast with elevated astrocytic calcium activity of high amplitude and low frequency (~ 0.005 Hz) reported in APP mice^55^. Nevertheless, aberrant calcium activity was consistent with the reports of disrupted astrocytic calcium signaling in mouse models of beta-amyloidosis^55,56^. Astrocytic calcium transients of APP mice exhibited maintenance of the slow-wave frequency but showed decreases in power starting at 3 months of age, before plaque deposition, yet when significant oligomeric Aβ is present in brain parenchyma^26^. This suggests that disruption of astrocytic calcium transients is an early event in the disease progression.

Since astrocytic calcium transients were disrupted, we used optogenetics to stimulate cortical astrocytes at the slow-wave frequency to allow the circuit to reach UP states of high power in APP mice. In doing so, we tested whether optogenetic targeting of astrocytes could restore slow waves. We showed that optogenetics allowed targeting of astrocytes with high fidelity and spatiotemporal precision within the intact cortico-thalamic circuit driving slow waves in vivo. Slow oscillations were restored by activation of ChR2-expressing astrocytes in APP mice. This suggests that even though slow oscillations are impaired, the structural integrity of the glial-neuronal circuit remains intact. At present, it is unclear whether optogenetic activation of astrocytes leads to restoration of global astrocyte calcium activity that propagates the slow wave signal. Alternatively, optogenetic activation of astrocytes could lead to restoration of local slow wave activity which then propagates via neurons. Finally, both neurons and astrocytes could participate in propagation of slow wave activity. APP mice exhibit deficit in NREM sleep during the dark cycle, but also during the light cycle^60^. Therefore, the optogenetic stimulation was not exclusive during the day or the night. Our study applied continuous optogenetic stimulation for 14–28 days without respect for natural wake/sleep states of the mouse. Future studies using closed-loop stimulation paradigm where stimulation is limited to bouts of NREM sleep will be necessary to address this limitation. Taken together with our earlier studies, it is clear that optogenetic stimulation of astrocytes as well as neurons leads to restoration of slow wave activity in APP/PS1 mice, suggesting that astrocytes interact with neurons when maintaining slow wave activity^24,26,27^.

APP mice overexpress a human mutant APP transgene and a human mutant PS1 variant, generate Aβ and deposit amyloid plaques starting at 4.5–5 months of age^66^. Accumulation of Aβ results in neuronal dysfunction and circuit impairments^6,67,68^. Here we showed that optogenetic targeting of astrocytes decreased the rate of amyloid beta deposition in APP mice. Xie et al.^58^ indicated that Aβ clearance can be improved via the glymphatic system during SWS. In addition, SWS, but not the total sleep time, showed a significant inverse correlation with the clearance of the amyloid beta in cognitively normal adults^69^. Future studies are necessary to determine whether optogenetic activation of astrocytes restores sleep in APP mice. Therefore, the reduction of amyloid beta deposition after stimulation of astrocytes might be enhanced via the glymphatic system due to improvements in sleep in APP mice. Since we found that optogenetic targeting of astrocytes restored SWA in APP mice, amyloid clearance via glymphatic pathway is a probable mechanism. Also, previous studies reported a link between Aβ production and SWA impairments in cognitively healthy adults^15,23,70^. Remarkably, increased Aβ deposition within medial prefrontal cortex has been associated with decreased SWA^23^. In addition, the levels of Aβ in cerebrospinal fluid increase after SWA suppression using auditory tones^70^. Our finding that restoration of astrocytic network function decreases amyloid plaque deposition serves to confirm and further expand on these previous results. It also strongly suggests that astrocytes regulate slow oscillations and participate in the pathogenesis of AD.

Amyloid beta disrupts neuronal calcium homeostasis which results in calcium elevations. Abnormal neuronal calcium homeostasis has been reported in AD^71–73^ and transgenic mouse models of beta-amyloidosis, such as APP and Tg2576 mice^26,62,74^. The measure of basal neuronal calcium has been adopted as a functional readout of treatment efficacy in animal models of AD^74–77^. Indeed, we saw neurons with calcium elevations or calcium overload in APP mice similar to earlier reports^26,27,62^. The toxicity of amyloid plaques or soluble oligomers surrounding plaques is responsible for the calcium overload in neurons, since the percentage of neurites exhibiting calcium overload is proportionally related to the proximity to plaques^62^. Furthermore, soluble oligomeric Aβ elicited pronounced calcium elevations in neurons in vivo^61,77^. Our findings indicate that neuronal calcium elevations in APP mice are prevented by activating astrocytes with optogenetics. Slow-wave restoration in this manner decreased deposition of amyloid plaques and as a consequence restored calcium homeostasis within neurons.

Deficits in SWA result in memory impairments^78,79^. Potentiation of SWA during sleep has been linked to improvements in declarative memory consolidation in human subjects^30,80–82^. Thus, SWA is necessary and sufficient for memory consolidation during sleep. Patients with Alzheimer exhibit sleep and memory impairments similar to APP mice^36,38^. Even though present study did not assess the effect of optogenetic stimulation on sleep in APP mice and future studies are needed to determine the effect of optogenetic stimulation on sleep–wake cycles of these mice, it did address the effect of optogenetic stimulation on sleep-dependent memory consolidation. The impairments in contextual fear memory start at 6 months of age^37^. Here we saw improvements of fear recall but not fear acquisition after optogenetic treatment. This suggests that aberrant astrocytic function actively contributes to deficits in sleep-dependent memory consolidation. Furthermore, these data provide further evidence that disrupted slow oscillations contribute to memory decline in AD. Optogenetic treatment of NTG mice failed to further improve their memory possibly due to a ceiling effect. It is unclear however whether the rescue of memory consolidation in APP mice was due to increased LFP power at 0.6 Hz during memory consolidation, due to enhanced amyloid beta clearance or both. Future studies testing the effect of optogenetic rescue on other types of memory including spatial memory will be needed.

At present, it is unclear whether the beneficial effects of optogenetic stimulation cease after light offset. Future experiments are needed to elucidate the potential of circuit entrainment beyond the stimulation period.

In summary, we demonstrate that astrocytes are critical for maintenance of proper circuit function in APP mice. Restoration of SWA via astrocyte targeting reduces amyloid plaque deposition, restores the calcium homeostasis in neurons, and improves sleep-dependent memory consolidation in APP mice. Our results provide evidence that dysfunctional slow oscillations underlie the memory impairments and pathophysiology of AD, as well as propose that targeting astrocytes to alter SWA might serve as a novel therapeutic strategy against AD.

## Materials and methods

### Experimental animals

The mouse experiments were performed with the approval of Massachusetts General Hospital (MGH) Institutional Animal Care and Use Committees (IACUC, protocol #2012N000085) for the use of experimental animals. All methods were carried out in accordance with relevant guidelines and regulations. All methods are reported in accordance with ARRIVE guidelines. The transgenic mouse line expressing the Swedish mutation of amyloid precursor protein (APP) and deltaE9 mutation in presenilin 1 (APPswe/PS1dE9; APP mice)^83^ and age matched non-transgenic littermate controls (NTG) were used in the study. Age and sex of animals were specified in methods and results section for individual experiments. Mice were housed 4 animals per cage in a pathogen-free environment with ad libitum access to food and water on a 12/12 h day and night cycle.

### Stereotaxic injection of adeno-associated viral vectors (AAVs)

Astrocytic calcium dynamics experiments employed 4–6 months-old APP and NTG mice. Animals were subjected to intracortical viral injections of 3 μl of AAV2/5-gfa2-YC3.6 targeting Yellow Cameleon 3.6 (YC3.6, titer = 4 × 10^12^ vg/ml) specifically to astrocytes in right somatosensory cortex with the following coordinates: anterior–posterior (AP) − 1.5 mm, lateral–medial (ML), − 1.5 mm; dorsal–ventral (DV) − 0.8 mm.

Acute optogenetics experiments used 4–6 months-old APP and NTG mice, that were injected with viral vectors targeting ChR2-mCherry or mCherry specifically to astrocytes (1.5 μl of AAV8-GFAP104-ChR2-mCherry, titer = 2.5 × 10^12^ mol/mL or 1.5 μl AAV8-GFAP104-mCherry, titer = 2.7 × 10^12^ mol/mL; UNC Vector Core) in the left frontal cortex (AP + 1 mm, ML + 1.5 mm, DV − 1 mm). In addition, viral vectors encoding Cre recombinase that targeted neurons (0.75 μl AAV9-hSyn-Cre, titer ≥ 1 × 10^13^ vg/mL, Addgene) and Cre-dependent voltage sensor Voltron (0.75 μl AAV1-hSyn-flex-Voltron-ST, titer ≥ 2 × 10^12^ vg/mL, Addgene) were injected into the right hemispheres of the same mice (AP − 3 mm, ML − 2 mm, DV − 0.2 mm) to monitor slow oscillations.

Chronic optogenetic experiments employed 8–10 months-old APP mice, that received stereotaxic injections of 1.5 μl of AAV8-GFAP104-ChR2-mCherry, titer = 2.5 × 10^12^ mol/mL or 1.5 μl AAV8-GFAP104-mCherry, titer = 2.7 × 10^12^ mol/mL into left frontal cortices (AP + 1 mm, ML + 1 mm, DV − 1 mm). Furthermore, 3 μl of AAV2-CBA-YC3.6 was injected into right somatosensory cortices (AP − 2 mm, ML − 1.5 mm, DV − 1 mm; titer = 2 × 10^12^ mol/mL) of same mice to allow monitoring neuronal calcium dynamics with multiphoton microscopy. Another cohort of NTG and APP mice that received stereotaxic injections of 1.5 μl of AAV8-GFAP104-ChR2-mCherry, titer = 2.5 × 10^12^ mol/mL or 1.5 μl AAV8-GFAP104-mCherry, titer = 2.7 × 10^12^ molecules/mL into left frontal cortices (AP + 1 mm, ML + 1 mm, DV − 1 mm) were subjected to open field and fear conditioning tests after receiving optogenetic treatment. Mice were handled 3 days before treatment.

All stereotaxic injections were performed according to the following procedure. The mice were anesthetized by inhalation of 1.75% isoflurane and placed on the stereotaxic instrument with a heating pad to maintain body temperature during the injections. Eye ointment was applied to the animals’ eyes prior to viral injections to prevent dryness. Fur was removed from the scalp with a trimmer. Liquid iodine was applied to the exposed skin and wiped clean with 70% isopropyl alcohol swabs 3 times each. An incision was made with sterile surgical scissors to expose the skull. A small burrhole was drilled over each injection site. Injections were performed with a 33-gauge metal needle and a 10 µl glass syringe, which was controlled by a stereotaxic injector. All the stereotaxic injections were performed at the rate of 100 nl/min. Following each intracortical injection, the scalp was sutured and animals were allowed to recover from anesthesia on a heating pad until awake and freely moving. At least 4 weeks were allowed for viral vector expression prior to imaging or optogenetic manipulation.

### Cranial window installation and multiphoton imaging

Four weeks after AAV2/5-gfa2-YC3.6 injection, a cranial window was implanted over the injection site in the right somatosensory cortex of each APP and NTG mouse to allow monitoring of astrocytic calcium transients with multiphoton microscopy. To achieve this, mice were anesthetized by inhalation of 1.75% isoflurane. A round craniotomy was made with a dental drill and a 5 mm glass coverslip was placed over the cortex. A mixture of dental cement and Krazy glue was used to secure the coverslip to the surrounding skull. A mode-locked titanium/sapphire laser (Mai Tai; Spectra-physics, Fremont, CA) generated two-photon fluorescence with 860 nm excitation, and three photomultiplier tubes (PMTs) (Hamamatsu Photonics, Japan) detectors collected the fluorescence emission in the range of 380–480 nm, 500–540 nm, and 560–650 nm. YC3.6-expressing astrocytes were imaged with a 25X water immersion objective (NA = 1.05, Olympus). Time-lapse images were acquired at a resolution of 256 × 256 pixels and sampling frequency at 2 μs/pixel. The power of the laser was kept below 50 mW at the objective to avoid phototoxicity. Astrocytic calcium transients and slow oscillations were monitored in anesthetized mice similar to other reports^53,57,84^.

The cohort of animals that underwent chronic optogenetic stimulation of ChR2-expressing astrocytes was first subjected to craniotomy with 5 mm cranial window implanted over the right somatosensory cortex. Z-stack images of amyloid plaque (Z-stack series 5 μm, 200–300 μm depth, cortical layer 2/3) and AAV2-CBA-YC3.6-expressing neurons (cell bodies and neuronal processes, neurites) (Z-stack series 2 μm, 200–300 μm depth, cortical layer 2/3) were acquired with a 25X water immersion objective at a resolution of 512 × 512 pixels and a sampling frequency at 4 μs/pixel. Methoxy-XO4 (10 mg/kg) was injected intraperitoneally a day before the imaging session to detect amyloid plaques.

### Optogenetic stimulation of ChR2-expressing astrocytes

Four weeks after ChR2-mCherry or mCherry alone injection in the left hemisphere and the voltage sensor Voltron injection in the right hemisphere, a small fluorophore with an appended HaloTag ligand Janelia Flour 525 dye (JF525 dye) (Janelia Research Campus, Ashburn, VA) was injected through the retro-orbital sinus a day before the imaging. JF525 dye was prepared by lyophilizing 100 nmol of JF525 dye and mixing in 20 μl of DMSO, 20 μl Pluronic F-127 (20% w/v in DMSO), and 100 μl of PBS. On the day of imaging, NTG and APP mice underwent cranial window installation to allow recording of slow oscillations in the right hemisphere. A 5 mm glass coverslip was placed over the right hemisphere. After the cranial window surgery, a fiber-optic cannula (length = 2 mm, Doric Lenses Inc, Quebec, Canada) was implanted over the ChR2-mCherry or mCherry injection site to activate astrocytes. Cannula tip was positioned above the cortex to prevent penetration and disruption of cortical circuit activity. The cannula and glass coverslip were secured with the dental cement mixture to the surrounding skull. The activity of neurons expressing Voltron labeled with JF525 was recorded with an upright fluorescence microscope with 2X objective (NA = 0.14, Olympus). An mKO/mOrange filter set (excitation: 530/30 nm, emission: 575/40 nm), and a 550LP dichroic mirror (49,014, Chroma Technology, Bellows Falls, VT) were used for fluorescence imaging of Voltron and JF525 expression. A CMOS camera (Hamamatsu Photonics, Japan) was used to collect images at 20 Hz. 3–5 trials of spontaneous slow oscillations were collected in the absence of optogenetic stimulation. Following these acquisitions, a fiber-optic patch cord (Doric Lenses Inc, Quebec, Canada) was attached to the cannula to activate ChR2-expressing astrocytes in left frontal cortex. 400 ms pulses of 473 nm light (3–6 mW) generated by a diode laser (Optoengine, Midvale, UT) were applied to the cortex at 0.6 Hz in APP mice and 1.2 Hz in NTG mice while recording slow oscillations. Replicates of 3–5 trials were acquired.

A separate cohort of mice underwent chronic light treatment to modulate the astrocytic network function. 400 ms pulses of 473 nm light (5–10 mW) were delivered at 0.6 Hz, 24 h/day for 14 or 28 days to restore the power of slow oscillations in the APP mice. Mice were housed in microdialysis bowls (Harvard Apparatus, Holliston, MA) with free access to food and water during entire optogenetic treatment. Following the chronic light treatment, mice were subjected to multiphoton imaging of amyloid plaques and YC3.6-expressing neurons as described above.

### Open field and contextual fear conditioning tests

Another cohort of NTG and APP mice expressing ChR2-mCherry or mCherry expression were subjected to open field and fear conditioning tests after receiving optogenetic treatment. Mice were handled 3 days before treatment. Following 13 days of chronic light treatment, the fiber-optic patch cords were removed from the mice to conduct the open field and fear conditioning tests. Mice were placed in a 27 cm × 27 cm × 27 cm arena one at a time and allowed to move freely for 10 min in a dark room during open field test. A camera recorded the locomotor activity of mice, and EthoVision XT software (Noldus, Wageningen, the Netherlands) was used to track and analyze the locomotor activity of APP mice.

Following the open field test, mice were placed in fear conditioning chambers (30 × 24 × 21 cm; MED-Associates, St. Albans, VT) one at a time and were monitored with a video camera for 5 min. Mice received 3 foot-shocks (1 s, 0.6 mA, 1 min interval) after 2 min of baseline recording. The conditioning chambers were surrounded with aluminum sides and a clear polycarbonate door. The foot-shocks were delivered with a removable grid floor that contained 36 stainless steel grid rods (3.18 mm in diameter, 8.13 mm apart). Following fear acquisition, mice were returned to the microdialysis bowls, their cannulas were connected to the fiber-optic patch cords for the last day of chronic light treatment. The day after fear acquisition, all mice were returned to the conditioning chambers for fear recall (recording for 3 min) in absence of foot-shocks. The threshold of freezing levels (i.e., the value of the motion index below which no movement is detectable) was determined. Fear responses measured as percentage of time spent freezing were determined using Video Freeze software (Med associates Inc, Fairfax, VT).

### Image analysis

The time-lapse images of cortical slow oscillations that were acquired with the CMOS camera were analyzed with ImageJ. The fluorescence intensity from Voltron_JF525 images were measured as the change in the pixel intensity over the baseline (ΔF/F0). Fourier transform analysis was used to quantify the ΔF/F0 traces of Voltron_JF525 images to determine the power of slow oscillations at 0.4–0.6 Hz, and the power of slow oscillations at 1.2 Hz when astrocytes were optogenetically activated at twice of the frequency.

ImageJ was used to process the time-lapse images of YC 3.6-expressing astrocytes and Z-stack images of YC 3.6-expressing neurites. YC 3.6 is a genetically encoded ratiometric calcium sensor. To determine YC3.6 ratio values, ratio images of YFP/CFP were created. Briefly, the images were preprocessed by subtracting the background from the last image of each volume from YFP and CFP channels. A median filter with a radius of 2 was applied to each channel. The regions of interest outlining the cell body of astrocytes or neurites were manually traced and selected using ImageJ. The YC3.6 ratios of the regions of interest (ROIs) were calculated by dividing the emitted fluorescence intensity of YFP by CFP. A custom-written Matlab script was used to create the pixel-by-pixel pseudocolored images based on the YFP/CFP ratios. Fourier transform analysis was performed to determine the power and frequency relationships of the calcium transients in astrocytes. We defined the number of astrocytes cycling at slow wave frequency as the power of the astrocytic calcium transient greater than three standard deviations above the mean.

Amyloid plaque image analysis was performed as follows. First, maximum intensity projections of each Z-stack volume were generated with Matlab. Then, ImageJ was used to manually select the ROIs for each plaque. Lastly, amyloid plaque number and amyloid plaque burden were counted and measured with the custom-written Matlab script. Data were presented as amyloid plaque numbers per cortical volume. Alternatively, amyloid plaque burden was calculated as percentage of cortical area occupied by amyloid. The z values of Z-stacks were comparable across conditions.

### Statistics

All the statistical analyses were performed using GraphPad Prism (version 6.01). Data was reported as mean ± SEM. Data sets were first tested with Shapiro–Wilk normality test, D’Agostino-Pearson omnibus normality test or Kolmogorov–Smirnov normality test. Normally distributed data was analyzed using student t test. Mann–Whitney test or Kruskal–Wallis test was used to analyze nonparametric data. *p < 0.05 was considered significant for 2 groups.

### Supplementary Information

Supplementary Information.

## Data Availability

All data generated or analyzed during this study are included in this published article (and its Supplementary Information files). Datasets not included are available from the corresponding author upon reasonable request.
