# Supplementary material for: Optogenetic targeting of astrocytes restores slow brain rhythm function and slows Alzheimer’s disease pathology
Source: Sci Rep. 2023 Aug 11;13:13075. doi: 10.1038/s41598-023-40402-3 (PMC10421876; doi:10.1038/s41598-023-40402-3)
Supplement: Supplementary file 1 — Supplementary Information. [file 41598_2023_40402_MOESM1_ESM.pdf]

**Supplemental information**

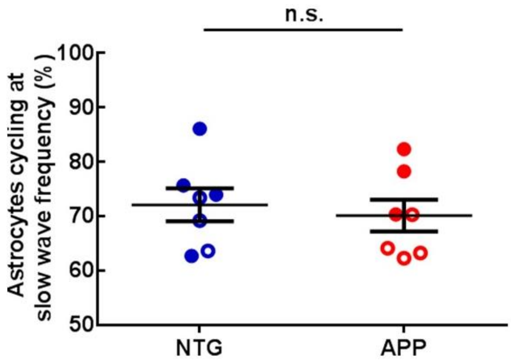


**Supplemental figure 1. The percentage of astrocytes cycling at the slow-wave frequency across genotypes.** The percentage of astrocytes that exhibited calcium transients at slow wave frequency (0.2-1 Hz) was comparable between NTG and APP groups. Males are represented by closed circles, and females by open circles. n.s. non-significant.

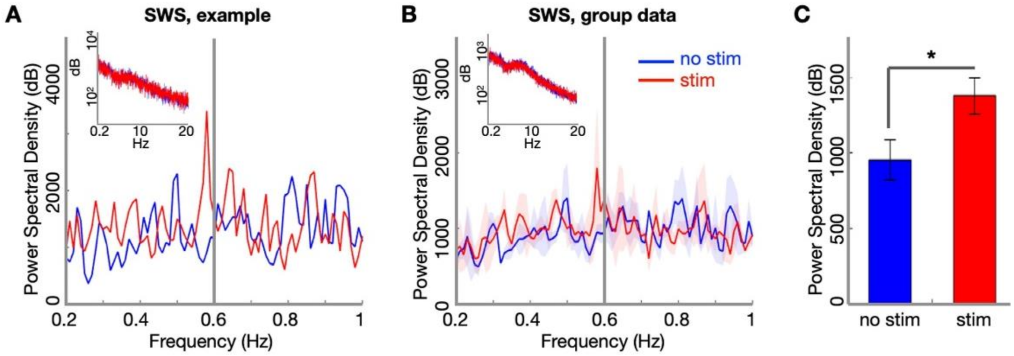


**Supplemental figure 2: Optogenetic stimulation of cortical astrocytes in slow-wave sleep is reflected in the cortical LFP in APP mice.** (A) LFP power spectral density (PSD) from one mouse. The vertical line (gray) identifies the stimulation frequency. Inset: PSD over 20 Hz. Blue: spontaneous LFP; Red: laser stimulation (0.6 Hz). (B) LFP power spectral density across the 3 APP mice. Inset: PSD over 20 Hz. (C) Group level effect of optogenetic stimulation on LFP power spectral density at 0.6 Hz. Data are shown as mean ± SEM (n = 3 mice, p = 0.030, 1-tailed paired t-test). Data in A and B are smoothed with a Gaussian window (σ = 0.03 Hz) for display purposes.

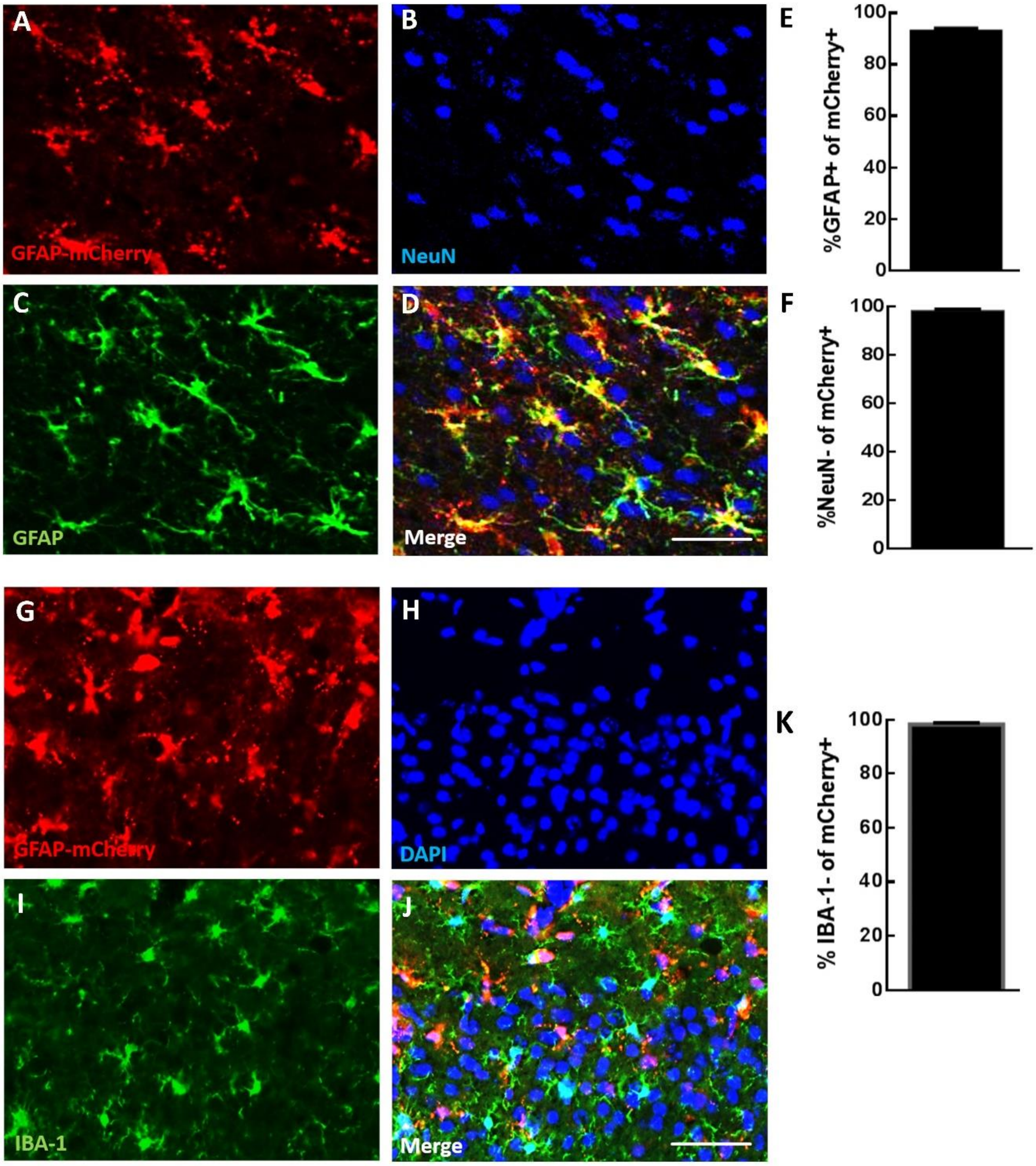


**Supplemental figure 3: GFAP promoter targets cortical astrocytes with high fidelity, not neurons or microglia.** (A, G) Representative images showing GFAP-mCherry expression in cortical astrocytes. (B, C) neuronal nuclei (NeuN) (B) and glial fibrillary acidic protein (GFAP) (C) expression following immunohistochemical staining. (D) Merge image A-C. (E, F) GFAP-mCherry was expressed in >92% of GFAP-expressing astrocytes. GFAP-mCherry-labeled astrocytes were mostly NeuN negative. (H, I) DAPI-labeled cells and microglia marker Iba-1 expression following immunohistochemical staining. (J) Merge image G-I. (K) GFAP-mCherry-labeled astrocytes were mostly IBA-1 negative. Values are mean ± SEM, Scale bar, 50μm.

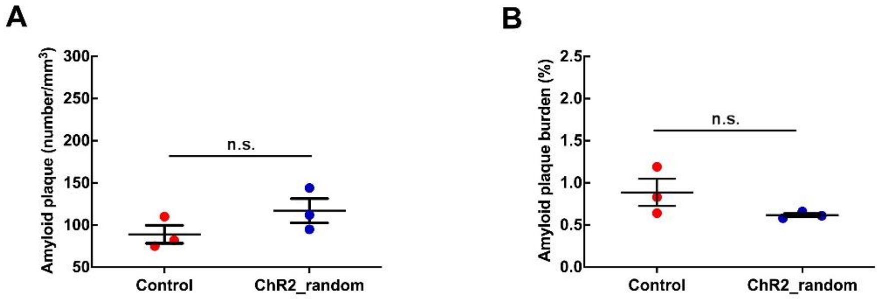


**Supplemental figure 4: Optogenetic stimulation of astrocytes at random frequency does not significantly alter amyloid plaque deposition in APP mice.** (A) Amyloid plaque number across conditions. (B) Amyloid plaque burden across conditions. Each point represents individual mouse. Values are presented as mean ± SEM. n.s. non-significant.

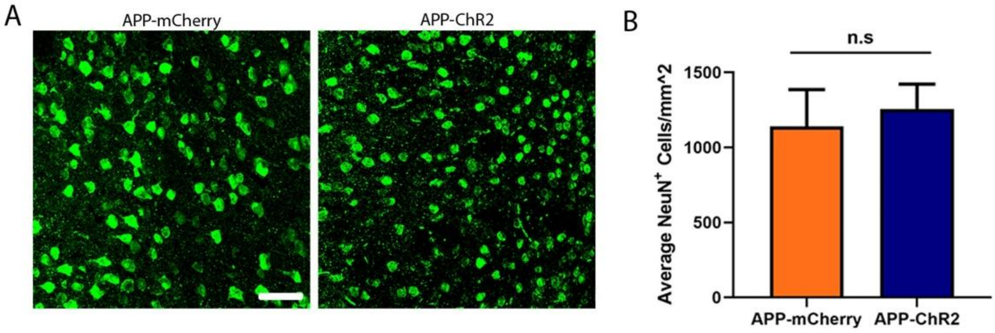


**Supplemental figure 5: Optogenetic stimulation of astrocytes does not result in neurodegeneration.** (A) Representative images of NeuN immunoreactivity in cortex of APP mice expressing mCherry (left) or ChR2 (right) treated with blue light for 2 weeks. (B) Bar graph showing optogenetic stimulation did not significantly alter the number of NeuN positive neurons in APP mice compared to light activation of mCherry. Scale bar, 50 µm. n.s. non-significant.

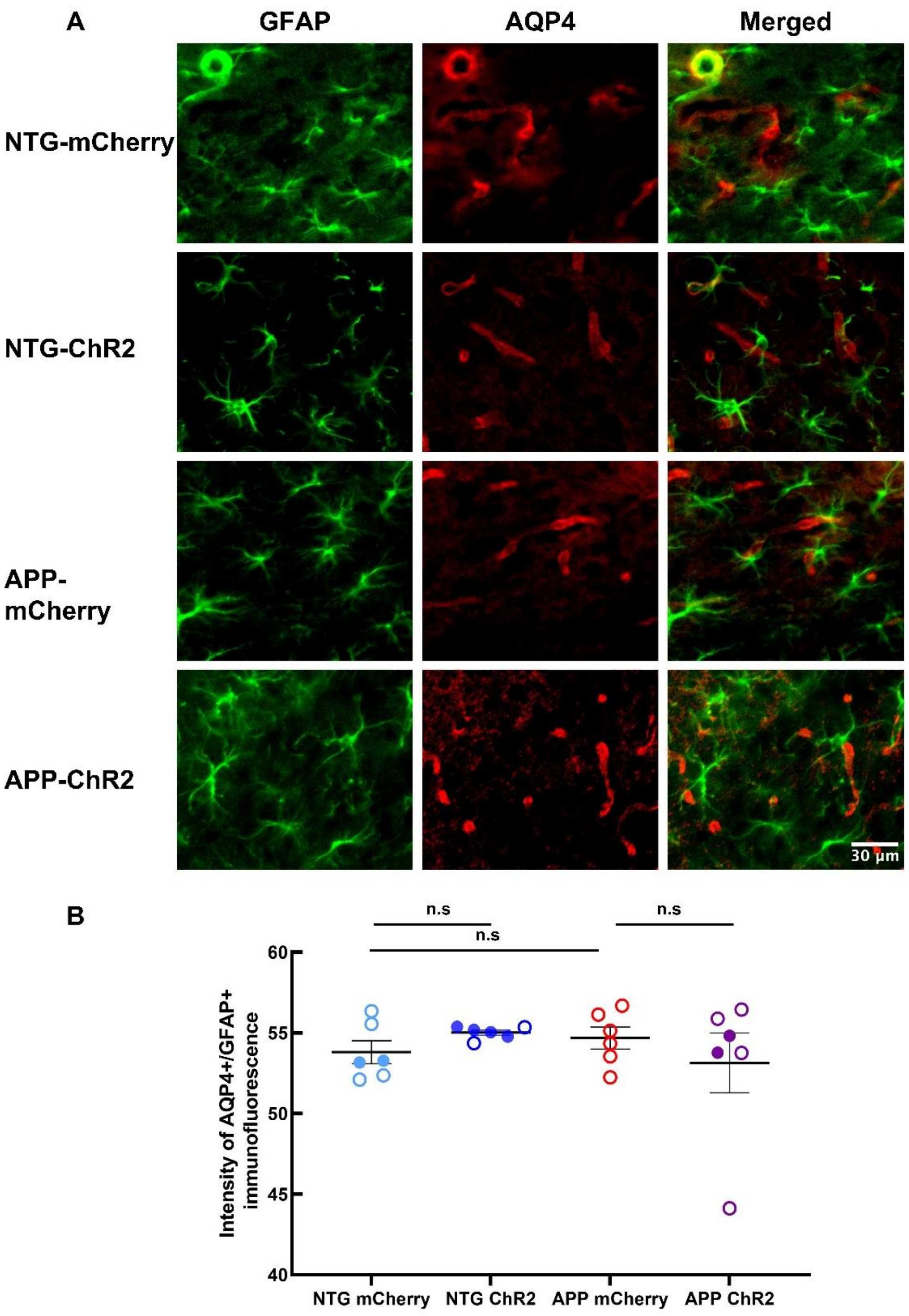


**Supplemental figure 6: The intensity of AQP4+ in GFAP+ astrocytes does not differ significantly after optogenetic stimulation of astrocytes.** (A) Representative images of GFAP and AQP4 immunoreactivity in cortex of NTG and APP mice expressing mCherry and ChR2. (B) The intensity of AQP4+/GFAP+ immunofluorescence across conditions. Each point represents individual mouse. Males are represented by closed circles, and females by open circles. Values are presented as mean ± SEM. n.s. non-significant.

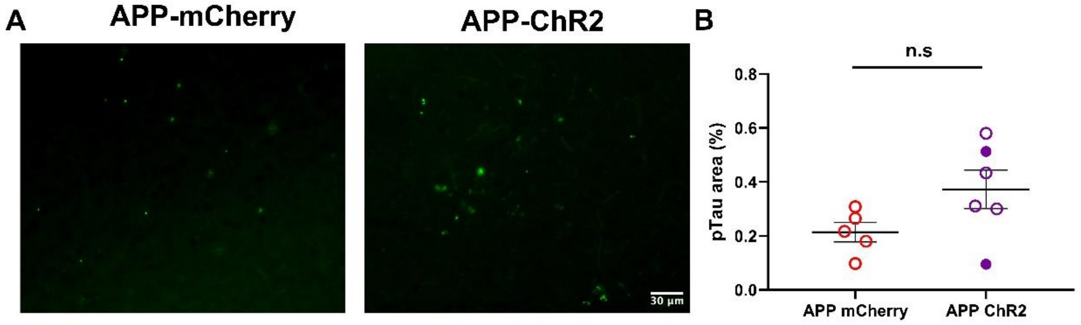


**Supplemental figure 7: The phospho-tau level was not altered significantly after activation of astrocytes by optogenetics in APP mice.** (A) Representative images showing the phospho-tau in cortex. (B) The percentages of area occupied by phospho-tau across conditions. Each point represents individual mouse. Males are represented by closed circles, and females by open circles. Values are presented as mean ± SEM. Scale bar, 30µm, n.s. non-significant.

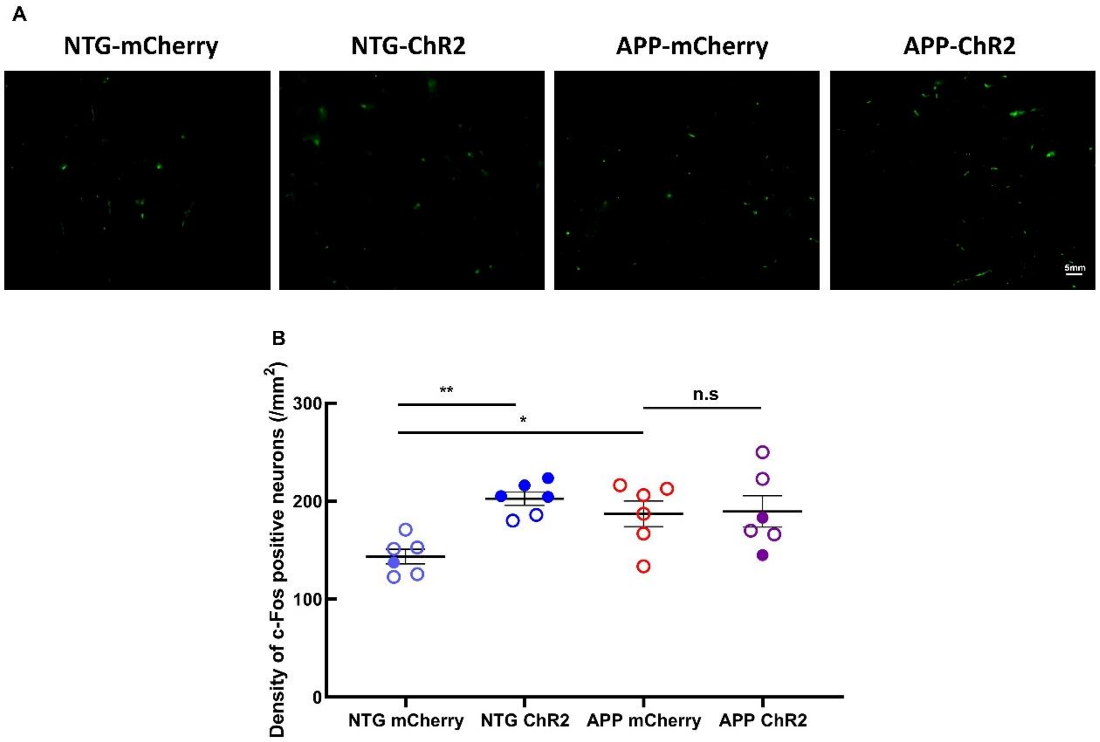


**Supplemental figure 8: Optogenetic stimulation of astrocytes did not significantly change the expression c-Fos in APP mice.** (A) Representative images of c-Fos immunoreactivity in cortex of NTG and APP mice expressing mCherry and ChR2. (B) A graph showing quantification of the density of c-Fos positive neurons in the cortex. Each point represents individual mouse. Males are represented by closed circles, and females by open circles. Values are presented as mean ± SEM. Scale bar, 5mm *p<0.05, **p<0.01, n.s. non-significant.

**Supplemental materials and methods**

**Immunohistochemistry**

The cohort of mice that underwent acute optogenetic stimulation expressing either ChR2-mCherry or mCherry empty viral specifically target to astrocytes was subjected to perform immunohistochemistry staining. The animals were euthanized with carbon dioxide inhalation and perfused transcardially with 1X phosphate-buffered saline (PBS). The left hemisphere that was injected with ChR2-mCherry or mCherry empty viral was harvested and fixed in 4% paraformaldehyde for 24 hours, then the brain was cryoprotected by 30% glycerol in 1X PBS. For visualizing viral infusion, the brain was cryosectioned into twenty-micrometer. The coronal sections of the brain were subjected to antigen retrieval in citrate buffer, then the sections were permeabilized with Triton X-100. Normal goat serum (NGS) was used to block non-specific antibody binding in tissues. The following primary antibodies that specifically target neuronal nuclei (NeuN; mouse monoclonal anti-NeuN, 1:500, MAB377; Millipore Sigma), glial fibrillary acidic protein (GFAP; rabbit polyclonal anti-GFAP, 1:1000; abcam ab7260 or 1:400, G3893 Millipore Sigma), ionized calcium binding adaptor molecule 1 (Iba-1; rabbit monoclonal anti-Iba1, 1:200; abcam ab178846), water channel Aquaporin-4 (AQP4; rabbit polyclonal anti-AQP4, 1:500, A5971 Millipore Sigma), phospho-Tau (AT8, mouse monoclonal anti-phophos tau, 1:400, MN1020B ThermoFisher), and rabbit monoclonal anti-c-Fos (1:500, 502047085 Fisher Scientific) were used in the study. The sections were incubated in primary antibody at 4°C overnight, then the sections were followed by incubation with the secondary antibodies, anti-mouse-pacific blue 405 (1:250) and anti-rabbit-Alexa fluor 488 (1:1000), respectively, for 1 h at room temperature. The sections were mounted with mounting media (Vectashield Antifade, Vector Laboratories) either with or without DAPI. Sections were examined with an inverted Zeiss microscope at 10X objective to identify the expressions. ROIs were drawn on the expression of mCherry, AQP4/GFAP, AT-8, and c-Fos in cortex. NeuN, GFAP, and Iba-1 expressing cells were manually identified using ImageJ. The percentage of GFAP-positive cells that colocalize with mCherry-positive cells were calculated. On the other hand, the percentage of NeuN and Iba-1-negative cells over mCherry-positive cells were presented in the figures. Furthermore, the intensity of AQP4+/GFAP+, pTau area, and the density of c-Fos positive neurons were presented in the figures. The sections from chronically treated APP mice (the hemispheres expressing mCherry or ChR2) were similarly processed for immunohistochemical analyses of NeuN.

**Electrophysiological recordings and analyses**

Four weeks after virus injection, an optical fiber was implanted at the virus injection site as described above and a custom-made adjustable probe consisting of 7 tungsten stereotrodes was implanted in the contralateral cortex (AP -2.5 mm, ML 2.5 mm from Bregma) and lowered into cortex. A skull screw was implanted over the cerebellum to serve as reference. After one week of recovery, electrophysiological recordings were acquired while the animal slept in the home cage. Local field potentials (LFP; 2 kHz per channel, 0.2-600 Hz) were recorded from each contact site on the probe (Neuralynx$^{TM}$). After a 30-min baseline recording, LFP was acquired during 30-min of laser illumination (473 nm, 0.6 Hz, 400 ms, 5-10 mW). For analyses, LFP data were downsampled to 1 kHz. Slow wave sleep (SWS) was classified by immobility (< 0.5 cm/sec for at least 3 minutes) in a sleep posture with delta/theta LFP power ratio > 2. LFP across the stereotrodes was reviewed and filtered in the slow oscillation range (0.2-1 Hz). A spectrogram from each stereotrode was calculated using the Chronux toolbox http://chronux.org/ with a moving window of size 2 s and step size 10

ms. A stereotrode with power at 0.6 Hz during optogenetic stimulation was selected for analysis. LFP power spectra were computed from 0.2 to 1 Hz with step size of 0.01 Hz and smoothed with a Gaussian window ($\sigma$=0.03 Hz). Power was compared across groups at the optogenetic stimulation frequency (0.60 Hz). Matlab (MathWorks, Natick, Massachusetts) was used for data analysis.

**Random stimulation**

Four weeks after ChR2-mCherry expression each APP animal (8 months old) received random stimulation with blue laser. Light pulses of fixed width were delivered according to a stochastic event-generating process approximating a stationary poisson process with a fixed intensity of 0.6 Hz. Treatment lasted 4 weeks. Amyloid plaques were imaged using multiphoton microscopy as described earlier. Amyloid plaque numbers and burden were compared to that of untreated APP mice of comparable age.
